# A Small Bowel Obstruction Secondary to Jejunal Bezoar

**DOI:** 10.7759/cureus.92257

**Published:** 2025-09-14

**Authors:** Georges Ziade, Naqqash Adnan, Zeeshan Khawaja

**Affiliations:** 1 Department of General Surgery, Royal Albert Edward Infirmary, Wigan, GBR; 2 Department of General Surgery, Wrightington, Wigan and Leigh NHS Foundation Trust, Wigan, GBR

**Keywords:** calcified bezoar, duodenal diverticula, exploratory laparotomy, gastrointestinal bezoar, jejunal diverticula, small bowel enterotomy, small-bowel obstruction

## Abstract

An 82-year-old woman presented with a two-day history of bilious vomiting, abdominal bloating, and epigastric discomfort. She remained hemodynamically stable without signs of peritonitis. Abdominal CT revealed small bowel obstruction with a transition point in the proximal jejunum, caused by a heterogeneous mass with curvilinear calcification, suspicious for a bezoar. Given its size, location, and the low likelihood of spontaneous passage, surgical intervention was undertaken. Laparotomy confirmed a 6×6 cm calcified bezoar located 70 cm distal to the duodenojejunal flexure, with multiple proximal jejunal and duodenal diverticula likely contributing to its formation. The bezoar was removed via enterotomy without complications. This case highlights an uncommon cause of small bowel obstruction and the importance of considering bezoars in elderly patients with underlying diverticular disease.

## Introduction

Small bowel obstruction (SBO) is a common surgical emergency, most frequently caused by postoperative adhesions, hernias, or malignancy. Bezoars represent an uncommon aetiology, responsible for approximately 2 to 4% of SBO cases [1,2]. Their occurrence in the proximal jejunum is rare, and even more so when associated with small bowel diverticula [3,4].

Bezoar formation is linked to several risk factors, including altered gastrointestinal anatomy, a history of abdominal surgery, poor mastication, and specific dietary habits [1,5]. Duodenal and jejunal diverticula, though often asymptomatic, can act as reservoirs for food debris and contribute to bezoar formation, which may lead to complications such as obstruction [6,7].

In our case, the patient presented with clinical and radiologic evidence of small bowel obstruction. CT imaging demonstrated a calcified bezoar at the proximal jejunum's transition point. Intraoperative findings confirmed multiple duodenal and jejunal diverticula. Surgical extraction via enterotomy was successful, and the patient had an uneventful recovery. This case underscores the importance of considering bezoars in elderly patients with small bowel obstruction, particularly those with underlying diverticular disease.

## Case presentation

An 82-year-old female with a Clinical Frailty Scale score of 5 presented with a two-day history of right-sided abdominal pain, bloating, and vomiting. She reported that the initial vomiting episode contained dark material, followed by two additional vomiting episodes at home. At presentation, she experienced abdominal distension but denied ongoing nausea, vomiting, or abdominal pain. Her last bowel movement and passage of flatus occurred the previous day.

The patient also mentioned that her daughter had been concerned about her reduced appetite and oral intake over the past three weeks. She described experiencing early satiety and a sensation of fullness after eating only small amounts. These symptoms prompted worries that she might not be maintaining adequate nutritional intake, although the patient denied significant weight loss or overt signs of malnutrition at presentation.

Her past medical history was significant for an open cholecystectomy with partial liver resection performed 32 years earlier, which had been converted from a laparoscopic approach due to adhesions. She also had a history of breast cancer that was managed with wide local excision, lymph node dissection, and radiotherapy. In addition, she suffered from trigeminal neuralgia and osteoarthritis of the hip, and had experienced previous episodes of pancreatitis.

She was functionally independent, living alone, and remained mobile, although her daughter recently provided her with a walking stick. She denied smoking and alcohol consumption. Her medications included atorvastatin and carbamazepine. She reported dizziness with previous use of co-codamol.

On arrival, her vital signs were largely within normal limits except for mild tachycardia (heart rate 108 bpm) (Table 1). Laboratory investigations revealed normal renal function, electrolytes, liver function tests, and amylase. Inflammatory markers were elevated, with a C-reactive protein of 38 mg/L. Notably, there was marked leucocytosis (36.3 ×10⁹/L) with neutrophilia and lymphocytosis (Table 2).

**Table 1:** Observations

Parameter	Measurement
Heart rate	108 beats/min
Blood pressure	129 / 86 mmHg
Oxygen saturation	99% on room air
Temperature	36.8°C

**Table 2:** Laboratory investigations

Test	Value	Reference Range
Urea	7.6 mmol/L	2.5–7.8 mmol/L
Creatinine	77 µmol/L	45–84 µmol/L
Sodium	132 mmol/L	135–145 mmol/L
Potassium	4.1 mmol/L	3.5–5.3 mmol/L
Estimated Glomerular Filtration Rate	62 mL/min/1.73m²	>90 mL/min/1.73m²
C-Reactive Protein	38 mg/L	<10 mg/L
Albumin	40 g/L	35–50 g/L
Alanine Aminotransferase	21 IU/L	7–35 IU/L
Alkaline Phosphatase	109 IU/L	30–130 IU/L
Bilirubin	8 µmol/L	3–21 µmol/L
Protein	59 g/L	60–80 g/L
Amylase	45 IU/L	20–100 IU/L
Haemoglobin	138 g/L	115–160 g/L
Red Blood Cell Count	4.51 x10¹²/L	3.8–5.2 x10¹²/L
Haematocrit	0.40 L/L	0.36–0.46 L/L
Mean Corpuscular Volume	89.0 fL	80–100 fL
Mean Corpuscular Haemoglobin	30.6 pg	27–32 pg
Red Cell Distribution Width	11.9%	11.5–14.5%
White Blood Cell Count	36.3 x10⁹/L	4.0–11.0 x10⁹/L
Neutrophils	7.9 x10⁹/L	2.0–7.5 x10⁹/L
Lymphocytes	26.6 x10⁹/L	1.0–4.0 x10⁹/L
Monocytes	1.7 x10⁹/L	0.2–0.8 x10⁹/L
Eosinophils	0.0 x10⁹/L	0.0–0.4 x10⁹/L
Basophils	0.1 x10⁹/L	0.0–0.1 x10⁹/L
Platelets	220 x10⁹/L	150–400 x10⁹/L
International Normalized Ratio	1.0	0.8–1.2
Prothrombin Time	11.2 seconds	10–12 seconds
Activated Partial Thromboplastin Time	18.2 seconds	25–35 seconds
Activated Partial Thromboplastin Time Ratio	0.7	0.8–1.2

Abdominal examination revealed mild distension of the lower abdomen with localized tenderness in the right periumbilical region, without rebound tenderness or signs of peritonitis. Hernial orifices were unremarkable. Digital rectal examination revealed soft stool, with no blood or palpable masses.

**Figure 1:**
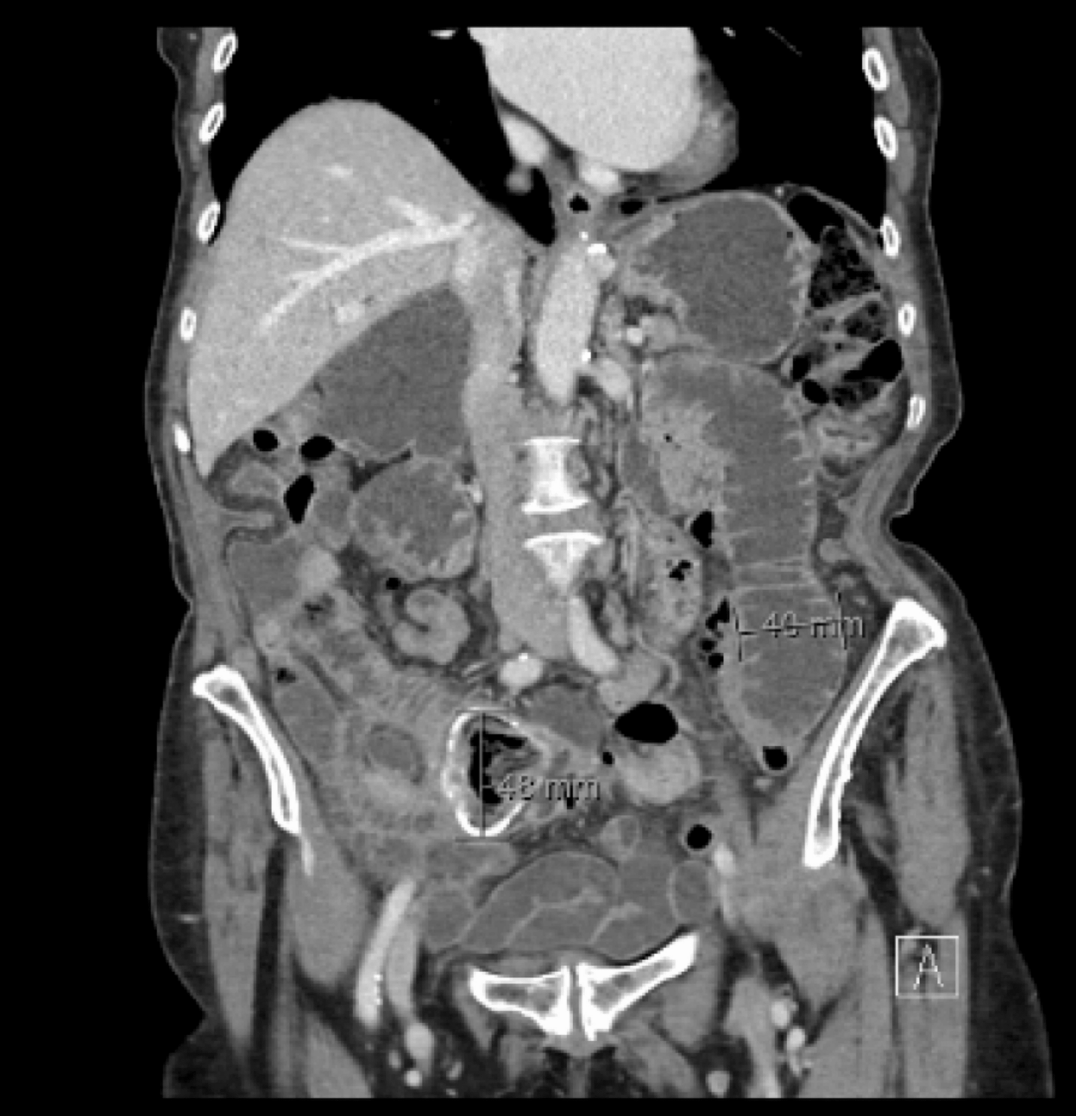
Coronal CT Coronal CT showing severely distended bowel loops and a heterogeneous structure with peripheral curvilinear calcification measuring up to 48 mm.

**Figure 2:**
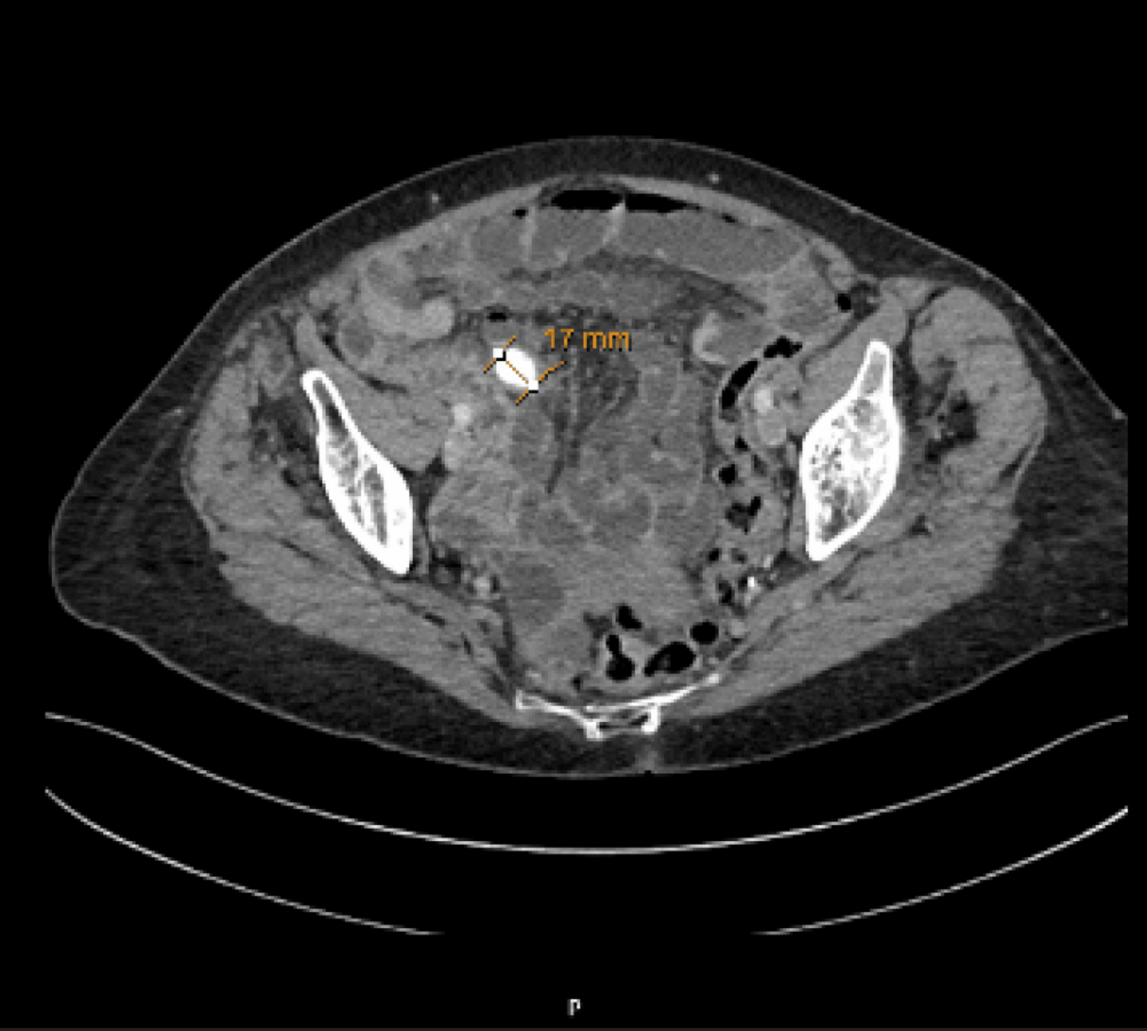
Axial CT Axial CT demonstrates a rounded, well-defined mass measuring 17 mm in diameter within the intestinal lumen.

Contrast-enhanced computed tomography (Figure 1, 2) of the abdomen and pelvis demonstrated significant small bowel distension with a transition point at the proximal jejunum. At this location, a heterogeneous intraluminal structure with peripheral curvilinear calcification was identified, suggestive of a bezoar or impacted food debris. Two large duodenal diverticula were noted, one occupying the gallbladder bed, along with scattered diverticula throughout the small and large bowel. No evidence of bowel perforation or free fluid was present. The liver, spleen, pancreas, kidneys, and adrenal glands were unremarkable, except for minor pancreatic atrophy and scattered renal cortical cysts. A small sliding hiatus hernia was also identified.

The patient was admitted under the surgical team with a provisional diagnosis of small bowel obstruction secondary to bezoar impaction. Initial management included nil by mouth, intravenous fluid resuscitation, nasogastric decompression via Ryle’s tube, urinary catheterisation, broad-spectrum intravenous antibiotics (piperacillin-tazobactam), venous thromboembolism prophylaxis, analgesia, and continuation of regular medications.

A detailed discussion was held with the patient and her family regarding the likely diagnosis and treatment options. Given the size and calcified nature of the bezoar seen on CT, we explained that spontaneous passage was unlikely, and that a trial of Gastrografin would likely not provide clinical benefit. We advised surgical intervention as the definitive treatment. We further explained that, given the patient’s frailty and advanced age, enterotomy and bezoar extraction was the preferred operative approach. The patient and her family understood the risks and benefits and were in agreement with the proposed plan.

The following day, the patient underwent an exploratory laparotomy. Intraoperatively, collapsed distal small bowel loops were identified, and a 6 × 6 cm hard, calcified bezoar was found in the mid-jejunum, approximately 70 cm distal to the duodenojejunal flexure. An enterotomy was performed over the lesion, and the bezoar was successfully extracted. The enterotomy site was closed with 2-0 polydioxanone (PDS) sutures in a seromuscular fashion. Multiple jejunal diverticula were noted, including a large proximal diverticulum likely contributing to bezoar formation. No other abnormalities were identified. The abdomen was closed in layers, and local anaesthetic was infiltrated into the wound. Blood loss was minimal (<100 mL), and a specimen of the bezoar was sent for analysis.

Postoperative care included continuation of the Ryle's tube on free drainage, nil by mouth status, ongoing antibiotics and analgesia, and re-initiation of thromboprophylaxis with low molecular weight heparin six hours postoperatively. The Ryle’s tube was spigotted after 24 hours, allowing the patient to take sips of water, and the tube was removed after 48 hours after confirming satisfactory oral tolerance. 

The histopathology report described the sample as an irregular piece of pale, firm material measuring 49 × 47 × 25 mm with a yellow, friable cut surface. Microscopic examination revealed acellular material composed predominantly of faecal matter with probable bacterial colonies present. The patient remained haemodynamically stable throughout her recovery and had an uneventful postoperative course. She was discharged without complications and did not require any further follow-up.

## Discussion

Bezoars are aggregates of indigestible material that accumulate within the gastrointestinal tract, most commonly in the stomach and small intestine, where anatomical narrowing and peristaltic forces, particularly at the ileocecal valve, predispose to impaction and obstruction [1-3]. They are classified based on their composition. The most common type is the phytobezoar, composed of indigestible plant fibres such as cellulose, lignin, and tannins. Phytobezoars are typically associated with high fibre diets and are often found in patients with impaired gastric motility or previous gastric surgery [1,2,5]. Trichobezoars, on the other hand, are formed from ingested hair, and are typically observed in young females with psychiatric disorders such as trichotillomania and trichophagia [8]. Lactobezoars are composed of inspissated milk or formula, and are seen almost exclusively in neonates, particularly premature infants with immature digestion [9]. Pharmacobezoars consist of concretions of undigested medications or drug delivery vehicles such as extended-release tablets, antacids, or enteric-coated pills [10]. Rarer types include metal bezoars and plastic bezoars, formed from the ingestion of foreign bodies, typically in individuals with pica or psychiatric illness [11].

The risk factors for bezoar formation in this patient included previous gastrointestinal surgery with altered anatomy, poor mastication, and the presence of jejunal diverticula. Several studies have identified jejunal diverticulosis as a potential nidus for bezoar development. These diverticula, often asymptomatic, can act as reservoirs for food debris, eventually leading to bezoar formation and potential complications such as obstruction or perforation. Bezoars are a rare but important cause of small bowel obstruction, accounting for approximately 2 to 4% of cases [4,6,7].

Radiologically, bezoars often present as well-defined intraluminal masses with a characteristic mottled gas appearance on CT, allowing differentiation from other causes of small bowel obstruction such as tumours, strictures, or adhesions [3]. Computed tomography is considered the most sensitive and accurate imaging modality for detecting bezoars, with reported sensitivities ranging from 73 to 95% [12] and high overall diagnostic accuracy of 83% [13]. In our patient, CT imaging demonstrated features of small bowel obstruction with a bezoar identified as a heterogeneous intraluminal structure with peripheral curvilinear calcification in the proximal jejunum. Although the classic mottled gas appearance is frequently described in the literature, it was not observed in this case [3,11]. Additionally, at least two large duodenal diverticula were noted arising from the second and third parts of the duodenum, with the proximal diverticulum occupying the gallbladder bed. During surgery, a calcified bezoar was found approximately 70 cm distal to the duodenojejunal flexure in the jejunum, adjacent to a prominent jejunal diverticulum, with further jejunal diverticula identified about 100 cm distal to the obstruction. While the duodenal diverticula were not directly implicated in bezoar formation, they may have contributed to altered motility or stasis. However, the large jejunal diverticulum near the bezoar was considered the most likely nidus for development. This case underscores the diagnostic value of CT imaging and the importance of correlating radiological findings with intraoperative anatomy to accurately determine the cause of obstruction. Although the patient was haemodynamically stable and showed no overt signs of peritonitis, the clinical presentation combined with imaging strongly suggested a mechanical obstruction unlikely to resolve with conservative management.

Conservative treatment options such as enzymatic dissolution, prokinetic agents, and endoscopic fragmentation are considered for gastric bezoars, but these approaches are generally ineffective for small bowel bezoars due to their distal location and the high risk of complete obstruction [2,5]. Consequently, surgical intervention remains the definitive treatment for small bowel bezoars, particularly in cases of large or impacted masses causing high-grade obstruction [14]. Surgical options include enterotomy with direct extraction of the bezoar, or in some cases, bowel resection if ischemia or necrosis is present [14]. Laparotomy has traditionally been the standard approach, allowing direct visualization and removal of the obstructing bezoar, as well as inspection for additional bezoars or complications [15]. More recently, laparoscopic techniques have been increasingly reported with favourable outcomes, offering advantages of reduced postoperative pain, shorter hospital stay, and quicker recovery, although laparoscopic management is dependent on surgeon expertise and patient factors [11]. In our case, after multidisciplinary discussion and consultation with the patient and her family, surgical intervention was advised because of the bezoar’s large size and calcification. The patient underwent exploratory laparotomy with enterotomy and successful bezoar extraction, followed by an uneventful recovery.

## Conclusions

Bezoars are rare but important causes of small bowel obstruction, particularly in patients with predisposing factors such as prior surgery or altered gastrointestinal motility. Prompt recognition on imaging and appropriate surgical management are essential to prevent complications. The presence of diverticula should also raise clinical suspicion for underlying causes of bezoar formation, especially when recurrent or large bezoars are encountered.
